# Consolidation Immunotherapy After Platinum-Based Chemoradiotherapy in Patients With Unresectable Stage III Non-Small Cell Lung Cancer—Cross-Sectional Study of Eligibility and Administration Rates

**DOI:** 10.3389/fonc.2020.586449

**Published:** 2020-12-01

**Authors:** Tanja Eichkorn, Farastuk Bozorgmehr, Sebastian Regnery, Lisa A. Dinges, Andreas Kudak, Nina Bougatf, Dorothea Weber, Petros Christopoulos, Thomas Muley, Sonja Kobinger, Laila König, Juliane Hörner-Rieber, Sebastian Adeberg, Claus Peter Heussel, Michael Thomas, Jürgen Debus, Rami A. El Shafie

**Affiliations:** ^1^ Department of Radiation Oncology, Heidelberg University Hospital, Heidelberg, Germany; ^2^ National Center for Radiation Oncology (NCRO), Heidelberg Institute for Radiation Oncology (HIRO), Heidelberg, Germany; ^3^ National Center for Tumor Diseases (NCT), Heidelberg University Hospital, Heidelberg, Germany; ^4^ Thoracic Clinic, Heidelberg University, Heidelberg, Germany; ^5^ Clinical Cooperation Unit Radiation Oncology (E050), German Cancer Research Center (DKFZ), Heidelberg, Germany; ^6^ Institute for Medical Biometrics and Informatics, Heidelberg University, Heidelberg, Germany; ^7^ Translational Lung Research Center Heidelberg (TLRC-H, German Center for Lung Research (DZL), Heidelberg, Germany; ^8^ Department of Diagnostic and Interventional Radiology, Thoracic Clinic, Heidelberg University, Heidelberg, Germany; ^9^ Department of Diagnostic and Interventional Radiology with Nuclear Medicine, Thoracic Clinic, Heidelberg University, Heidelberg, Germany; ^10^ German Cancer Consortium (DKTK), Partner Site Heidelberg, German Cancer Research Center (DKFZ), Heidelberg, Germany

**Keywords:** durvalumab eligibility rate, durvalumab admission rate, PACIFIC criteria, non-small cell lung cancer stage III, definitive platinum-based chemoradiotherapy

## Abstract

**Introduction:**

The PACIFC trial demonstrated a significant benefit of durvalumab consolidation immunotherapy (CIT) after definitive platinum-based chemoradiotherapy (P-CRT) for survival in stage III non-small cell lung cancer (NSCLC). It is unknown how many patients are eligible in clinical practice to receive CIT according to PACIFIC criteria compared to real administration rates and what influencing factors are.

**Patients and Methods:**

We analyzed 442 patients with unresectable stage III NSCLC who received P-CRT between 2009 and 2019 regarding CIT eligibility rates according to PACIFIC criteria and administration rates since drug approval.

**Results:**

Sixty-four percent of 437 patients were male, median age was 63 years [interquartile range (IQR): 57–69]. The most common histologic subtypes were adenocarcinoma (42.8%) and squamous cell carcinoma (41.1%), most tumors were in stage IIIB (56.8%). Mean PD-L1 tumor proportion score (TPS) was 29.8% (IQR: 1–60). The median total RT dose was 60 Gy (IQR: 60–66). Platinum component of P-CRT was evenly distributed between cisplatin (51.4%) and carboplatin (48.6%). 50.3% of patients were eligible for CIT according to PACIFIC criteria. Observed contraindications were progressive disease according to RECIST (32.4%), followed by a PD-L1 TPS < 1% (22.3%), pneumonitis CTCAE ≥ 2 (12.6%) and others (4.9%). One year after drug approval, 85.6% of patients who were eligible according to PACIFIC criteria actually received CIT. Time interval between chemotherapy start and radiation therapy start (OR 0.9, 95% CI: [0.9; 1.0] p = 0.009) and probably cisplatin as platinum-component of P-CRT (OR 1.5, 95% CI: [1.0; 2.4] p < 0.061) influence CIT eligibility. Highly positive PD-L1 TPS (≥50%; (OR 2.4, 95% CI: [1.3; 4.5] p = 0.004) was associated to a better chance for CIT eligibility.

**Conclusion:**

Eighty-five percent of potentially eligible patients received CIT one year after drug approval. Fifty percent of patients did not meet PACIFIC criteria for durvalumab eligibility, this was mainly caused by disease progression during platinum-based CRT, followed by therapy-related pneumonitis and PD-L1 TPS < 1% (in view of the EMA drug approval).

## Introduction

Lung cancer is an important challenge for public health due to its frequency (1) and poor prognosis (2). Non-small cell lung cancer (NSCLC) is the most common pulmonary malignancy and one third of patients primarily diagnosed with NSCLC are in a locally advanced stage without distant metastasis, defined as stage III. Despite effort was taken in the past decades to investigate treatment options for unresectable stage III NSCLC, definitive platinum-based doublet chemoradiotherapy (P-CRT) merely demonstrated a 5-year survival rate of 15% and a median progression-free survival of 8 months (2, 3).

Therefore, a new milestone was reached when results from the PACIFIC trial were first published in 2017, that investigated a human monoclonal antibody against programmed death-ligand 1 (PD-L1) as consolidation therapy after completed definitive P-CRT in unresectable stage III NSCLC. The data clearly demonstrated a significant improvement of overall survival as well as progression-free survival without major safety concerns. The median progression-free survival significantly increased with durvalumab consolidation immunotherapy (CIT) after P-CRT to 16.8 months versus 5.6 months with placebo (4).

However, the PACIFIC trial was conducted on a very specific sub-collective due to strict inclusion criteria. The characteristics of this highly selected patient collective are arguably discordant with the perceived clinical reality of stage III NSCLC patients, as a small retrospective analysis recently demonstrated. It reports that merely two thirds of the 73 patients included were eligible for CIT according to the three-criteria disease progression, therapy-related toxicity, and WHO performance status (5). Nevertheless, due to the small size of the analyzed patient collective the few criteria investigated and the purely descriptive nature of the analysis, reliable data on subject of CIT eligibility is still lacking. Furthermore, predictive factors for this endpoint have not been analyzed.

It is therefore the purpose of this analysis to describe the rate of CIT eligibility according to the PACIFIC criteria in a large cohort of consecutive stage III NSCLC patients treated at a European tertiary cancer center over a 10-year period. Furthermore, predictive factors that could possibly influence upfront treatment decisions are analyzed. Additionally, for the first time this project compares the rates of durvalumab eligibility with de facto rates of CIT administration since drug approval in the analyzed collective.

## Patients and Methods

Four hundred thirty-seven patients with unresectable stage III NSCLC who received P-CRT between 2009 and 2019 at a European comprehensive cancer center were included in this analysis. Patient and treatment data were extracted from a clinical database maintained at our institution and from medical and official records. Primary endpoint was durvalumab eligibility defined as meeting the PACIFIC criteria. These criteria included among others patients with an unresectable stage III NSCLC and a PD-L1 positivity of ≥ 1% who had received definitive P-CRT with at least two cycles of platinum-based chemotherapy, a high performance status defined by ECOG 0-1, the absence of unresolved toxic effects of Common Terminology Criteria for Adverse Events (CTCAE) ≥ 2, the absence of disease progression after P-CRT and the absence of other general contraindications against immunotherapy. To check for unresolved toxic effects according to the National Cancers Institute’s CTCAE ≥ 2 (version 4.03), both medical records and imaging reports were analyzed. Disease progression was assessed by reviewing CT imaging reports. The first CT imaging follow-up study after completed P-CRT was used and compared to the last imaging study before CRT. Tumor response was evaluated by “Response Evaluation Criteria in Solid Tumors” (RECIST) version 1.1 and divided into complete or partial response, stable disease and progressive disease (6, 7). Rates of patients who actually received CIT after P-CRT in clinical practice since drug approval in the European Union on 21st September 2018 were assessed reviewing the patient files.

All analyses were performed following institutional guidelines and the Declaration of Helsinki of 1975 in its most recent version. Ethics approval for the study was granted by the Heidelberg University ethics committee on November 20^th^, 2019 (#S-767/2019). Patient confidentiality was maintained by anonymizing patient data to remove any identifying information.

### Statistical Analysis

Descriptive statistics for baseline variables (**Tables 1** and **2**) and for endpoints (**Table 3**) include means (SD) and/or median (IQR and range, as appropriate) for continuous variables and absolute and relative frequencies for categorical variables. To identify influencing factors on durvalumab eligibility a logistic regression model for durvalumab eligibility with a stepwise (forward/backward) variable selection procedure by Akaike information criterion (AIC) was used. To verify the variable selection results a bootstrap approach using 200 bootstrap samples was conducted. Gender (male versus female), time interval from chemotherapy start until radiation therapy start (in weeks) and platinum component of platinum-based chemotherapy (cisplatin versus carboplatin) are selected by the stepwise procedure. Results of logistic regression analysis are presented as Odds Ratio (OR), the corresponding confidence interval (CI), and p-value. Regression analysis was conducted for the entire collective as well as the subcollective with data on PD-L1 expression available. In the subcollective, the logistic regression model includes categories of PD-L1 tumor proportion score (TPS <50% versus ≥50%) as additional covariate. Since this is a retrospective exploratory data analysis, p-values are of descriptive nature. Statistical analyses are performed with the software *R Version 3.6.2*.

**Table 1:** Patient baseline characteristics.

	n = 437 [%]	
**Age at initial diagnosis (years)**		
mean	63.3	
median	63	
standard deviation	8.3	
quartile 1–quartile 3	57–69	
minimum-maximum	34–83	
**Gender**		
female	158	[36.2%]
male	279	[63.8%]
**Risk factors**		
smoker	184	[42.1%]
asbestos exposition	23	[5.3%]
previous malignoma	65	[14.9%]
**T stage**		
1	54	[12.4%]
2	98	[22.4%]
3	103	[23.3%]
4	183	[41.9%]
**N stage**		
0	7	[1.6%]
1	26	[6.0%]
2	229	[51.7%]
3	178	[40.7%]
**Stage**		
III A	142	[32.5%]
III B	248	[56.8%]
III C	47	[10.8%]
**Primary histology**		
adenocarcinoma	187	[42.8]
acinar	3	[0.7%]
papillary	5	[1.1%]
solid	9	[2.1%]
mixed subtype	6	[1.4%]
not specified	164	[37.5%]
adenosquamous carcinoma	3	[0.7%]
basal cell carcinoma	1	[0.2%]
large cell carcinoma	15	[3.4]
neuroendocrine	11	[2.5%]
mixed type	3	[0.7%]
not specified	1	[0.2%]
mixed small cell carcinoma	9	[2.1%]
pleomorphic carcinoma	1	[0.2%]
spindle cell carcinoma	1	[0.2%]
squamous cell carcinoma	180	[41.1]
basaloid	11	[2.5%]
papillary	1	[0.2%]
not specified	168	[38.4%]
unknown subtype	40	[9.2%]
**PD-L1**		
mean	29.8	
median	10	
standard deviation	n.a.	
quartile 1–quartile 3	1–60	
minimum – maximum	0–100	
**PD-L1 expression**		
PD-L1 available	190	[43.5%]
0%	43	[22.6%]
1-20%	75	[39.5%]
>20%	72	[37.9%]
≥50%	58	[30.5%]
missing	247	[56.5]
**WHO performance status (ECOG)**		
0	244	[55.8%]
1	189	[43.3%]
2	4	[0.9%]
3	0	[0.0%]
4	0	[0.0%]
5	0	[0.0%]

**Table 2:** Treatment characteristics.

	n = 437 [%]	
**Time primary diagnosis until radiation therapy start (weeks)**		
mean	11.1	
median	10	
standard deviation	6.9	
quartile 1–quartile 3	5.3–16.1	
minimum-maximum	0–33	
**Time chemotherapy start until radiation therapy start (weeks)**		
mean	7.2	
median	5.5	
standard deviation	6.2	
quartile 1–quartile 3	2–12	
minimum-maximum	0–26.9	
**Time primary diagnosis until radiochemotherapy end (weeks)**		
mean	17.4	
median	16.3	
standard deviation	7.2	
quartile 1–quartile 3	11.9–22.6	
minimum-maximum	0–40	
**Time radiochemotherapy end until first imaging follow-up (weeks)**		
mean	3.8	
median	3.4	
standard deviation	n.a.	
quartile 1–quartile 3	0–5.7	
minimum-maximum	0–16.3	
**Specification of radiochemotherapy end**		
regular end	387	[88.6%]
regular dose	362	[82.8%]
dose reduction	25	[5.7%]
premature end	50	[11.4%]
therapy related toxicity	14	[3.2%]
disease progression	15	[3.4%]
other reasons	21	[4.8%]
**Major interruptions of radiation therapy (>3 days)**		
no	319	[73.0%]
yes	118	[27.0%]
**Duration interruptions of radiation therapy (days)**		
mean	2.7	
median	2	
standard deviation	3.21	
quartile 1–quartile 3	0–4	
minimum-maximum	0–9	
**Radiotherapy total dose (cGy)**		
mean	6198	
median	6000	
standard deviation	321.7	
quartile 1–quartile 3	6,000–6,600	
minimum-maximum	5,400–6,600	
**Platinum-based chemotherapy**		
Cisplatin	223	[51.4%]
Carboplatin	211	[48.6%]
**Platinum-based chemotherapy combination partner**		
Alimta	8	[1.8%]
Docetaxel	1	[0.2%]
Etoposide	18	[4.1%]
Gemcitabine	25	[5.7%]
nab-Paclitaxel	1	[0.2%]
Paclitaxel	9	[2.1%]
Permetrexed	3	[0.7%]
Vincristin	1	[0.2%]
Vinorelbine	371	[84.9]
**Number of platinum-based chemotherapy cycles**		
mean	2.93	
median	3	
standard deviation	1.19	
quartile 1–quartile 3	2–4	
minimum-maximum	0–8	

**Table 3:** Durvalumab eligibility according to PACIFIC criteria.

	n = 437 [%]	
**PD-L1 expression (n = 193 since testing implemented)**		
0%	43	[22.3%]
1–100%	147	[77.7%]
**Initial response to platinum-based radiochemotherapy**		
non-progressive disease	288	[67.6%]
complete or partial remission	195	[45.8%]
stable disease	93	[21.8%]
progressive disease	138	[32.4%]
**Therapy-related toxicity**		
pneumonitis	65	[14.9%]
CTCAE °1	3	[0.7%]
CTCAE °2	52	[11.9%]
CTCAE °3	6	[1.4%]
CTCAE °4	4	[0.9%]
CTCAE °5	0	[0%]
other toxicities	0	[0%]
**General condition**		
WHO performance status (ECOG) <2	433	[99.1%]
WHO performance status (ECOG) ≥2	4	[0.9%]
**Number of platinum-based chemotherapy cycles**		
≥2 cycles	435	[99.5%]
<2 cycles	2	[0.5%]
**Other contraindications**		
h/o immunotherapy	0	[0%]
h/o other study medications	0	[0%]
h/o immunodeficiency	0	[0%]
h/o autoimmune disease	15	[3.5%]
h/o rheumatoid arthritis	9	[2.1%]
h/o other autoimmune disease	6	[1.4%]
h/o uncontrolled comorbidity	1	[0.0%]
**Overall CIT eligibility**		
no	217	[49.7%]
yes	220	[50.3%]

CTCAE, Common Terminology Criteria for Adverse Events.

## Results

### Patient and Treatment Characteristics

Median patient age at the beginning of radiotherapy (RT) was 63 years (IQR: 57–69) and two thirds were male. The most common histology was adenocarcinoma with 42.8%, directly followed by squamous cell carcinoma with 41.1%, which was prevalent with nearly the same frequency. Other histologic subgroups were rare. In 9.2%, the NSCLC subtype was unknown. Nearly half of patients (42.1%) were smokers. Most patients with stage III NSCLC were diagnosed with an advanced T stage (T4 in 42.9%) and an intermediate or advanced N stage (N2 in 51.7%; N3 in 40.7%) and therefore NSCLC stage IIIB was the most common subtype. Data regarding PD-L1 expression were available in nearly half of the patients (43.5%). This subgroup consists mainly of those patients treated after the European Medical Association’s (EMA) approval of CIT, which specifies a condition of at least 1% PD-L1 expression and which made PD-L1 expression analysis mandatory in stage III patients. Mean PD-L1 positivity was 29.8% (IQR: 1–60). WHO performance status (ECOG) was 0 or 1 in most patients.

A median period of 10.0 weeks (IQR: 5.3–16.1) passed after primary diagnosis until patients received the first fraction of radiation therapy simultaneous to chemotherapy. In the majority of patients, CRT was administered sequentially with chemotherapy starting first and RT following within a median period of 5.5 weeks (IQR: 2.0–12.0). Therefore, it took a median period of one month from primary diagnosis until administration of the first cycle of chemotherapy. Within a median period of four months (16.3 weeks, IQR: 11.9–22.6) after primary diagnosis, definitive simultaneous CRT was completed. A median period of one month (3.4 weeks, IQR: 0–5.7) after CRT finished the first imaging follow-up took place. In most cases (88.6%), CRT ended regularly even if few patients (5.7%) needed a dose reduction. In 11.4% of patients, CRT was stopped prematurely due to disease progression (3.4%), treatment-related toxicity (3.2%), or other reasons (4.8%). When radiation therapy was interrupted, interruptions lasted a median period of two days (IQR: 0–4). Major interruptions of radiation therapy defined by interruptions of more than three days were observed in 27.0% of patients. The median total RT dose was 60 Gy (IQR: 60–66). Platinum component of P-CRT was evenly distributed between cisplatin (51.4%) and carboplatin (48.6%). Chemotherapy combination partner was in most cases vinorelbine (84.4%), followed by gemcitabine (5.7%). Gemcitabine was used as a chemotherapy combination partner up to the early 2010’s as alternative institutional standard or as an alternative for patients with polyneuropathy. In very rare occasions a downstaging led to gemcitabine administration. A median of 3.0 cycles of chemotherapy (IQR: 2.0–4.0) were administered. Detailed patient characteristics are presented in **Table 1**, detailed treatment characteristics are presented in **Table 2**.

### Durvalumab Eligibility

Results of descriptive analysis of PACIFIC criteria for durvalumab eligibility are demonstrated in **Table 3**. A total of 50.3% of patients in the study cohort fulfilled all eligibility criteria to receive CIT. Contraindications for durvalumab eligibility according to the PACIFIC criteria were an insufficient initial response to P-CRT, defined as progressive disease according to RECIST (32.4%), followed by a PD-L1 positivity of 0% (22.3% of available PD-L1 data). In addition, 5.5% of all patients were not eligible for durvalumab consolidation of only PD-L1 <1% as the very only reason for exclusion. An increased therapy-related toxicity was the third most common reason for durvalumab ineligibility (14.2%). In all cases, increased therapy-related toxicity was due to pneumonitis CTCAE ≥ 2. In rare occasions a bad general condition (0.9%) or an insufficient number of P-CRT cycles led to ineligibility for CIT. In this cohort, 3.5% of patient had a documented other contraindication for CIT, most frequently a rheumatoid arthritis. Mild autoimmune diseases without need for therapy like a mild psoriasis were not excluded from CIT.

Descriptive analysis of the number of patients receiving CIT after P-CRT since drug approval in European Union at 21st September 2018 demonstrated that rates were continuously rising. From 21st September 2018 to the 31st December 2019, the overall rate of patients who received CIT rose to 50.0% (19 of 38 patients) and in conclusion 85.6% of patients who were eligible due to PACIFIC criteria really received CIT about 1 year after drug approval.

**Figure 1:**
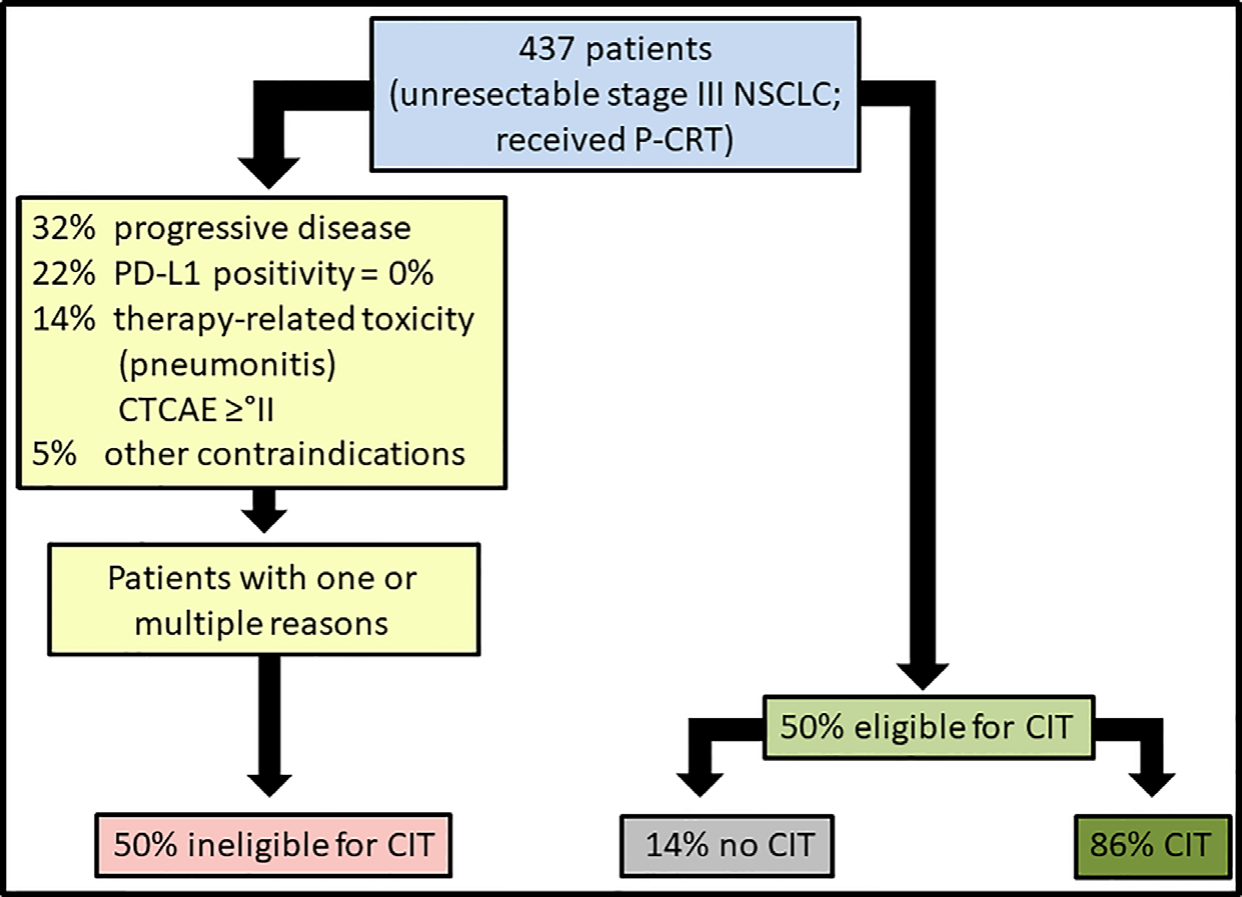
Eligibility and administration rates of consolidation immunotherapy (CIT) with durvalumab according to PACIFIC criteria. CIT, consolidation immunotherapy; CTCAE, Common Terminology Criteria for Adverse Events; NSCLC, non-small cell lung cancer; P-CRT, definitive platinum-based chemoradiotherapy; PD-L1, programmed death-ligand 1.

Results of explorative analysis of factors contributing to durvalumab eligibility are presented in **Table 4**. In part A, the entire cohort was included in a logistic regression analysis with durvalumab eligibility as dependent variable and multiple covariates as independent variables. In part B, the same analysis was performed only for the subcohort with available PD-L1 expression. Factors considerably influencing durvalumab eligibility were a shorter time interval between chemotherapy start and radiation therapy start (OR 0.9, 95% CI: [0.9; 1.0] p = 0.009) and probably cisplatin as platinum-component of P-CRT (OR 1.5, 95% CI: [1.0; 2.4] p = 0.061) influence CIT eligibility. Beyond the minimum required level of 1%, PD-L1 expression did influence durvalumab eligibility. Highly positive PD-L1 TPS (≥50%; (OR 2.4, 95% CI: [1.3; 4.5] p = 0.004) was associated to a better chance for CIT eligibility. **Figure 1** illustrates a flowchart of CIT eligibility and CIT administration according to PACIFIC criteria.

**Table 4:** Factors significant in logistic regression analysis and bootstrap analysis for the endpoint of durvalumab eligibility with corresponding odds ratios and p-values.

A) Analysis for entire cohort (n = 434)					
	Odds Ratio	95% Confidence Interval			p-value
**Durvalumab eligibility**					
gender (male)	0.71	0.47	–	1.07	**0.1**
time chemotherapy start until radiation therapy start (weeks)	0.96	0.93	–	0.99	**0.009**
platinum-based chemotherapy (Cisplatin)	1.53	0.98	–	2.38	0.061
**B) Analysis for subcohort with PD-L1 data available (n = 190)**					
	**Odds Ratio**	**95% Confidence Interval**			**p-value**
**Durvalumab eligibility**					
gender (male)	0.71	0.38	–	1.32	**0.278**
time chemotherapy start until radiation therapy start (weeks)	0.99	0.94	–	1.04	**0.642**
platinum-based chemotherapy (Cisplatin)	1.38	0.70	–	2.75	0.354
PD-L1 (≥50%)	2.40	1.30	–	4.52	**0.006**

Time intervals are tested longer versus shorter. P-values ≤ 0.05 were printed in bold.

## Discussion

We investigated how many patients in clinical practice are eligible to receive CIT to potentially benefit from the significant survival benefits shown in the PACIFIC trial.

Between 2009 and 2019, 50.3% of our 437 patients that received platinum-based definitive CRT for NSCLC sage III fulfilled the PACIFIC criteria for CIT after P-CRT. In the recently treated subgroup with PD-L1 expression results available, this ratio was 72%, considering the additional requirement of a minimum 1% PD-L1 expression. Compared to that, a study that analyzed data of 81 patients from January 2011 to May 2018 published an eligibility rate of approximately 70% without considering PD-L1 expression levels (5).

In addition to eligibility rates the presented study is able to compare them to real durvalumab administration rates at a large European university hospital. Recently published data demonstrated that real-world end points are consistent with outcomes observed in randomized clinical trials among immunotherapy-treated patients with advanced NSCLC (8). Durvalumab CIT in patients with NSCLC stage III was approved by the EMA on September 21st 2018. Since then, durvalumab administration rates continuously rose and reached about a rate of 50% of all patients with NSCLC stage III 1 year after drug approval, amounting for 85% of the patients eligible according to the PACIFIC criteria. This development seems to be reasonable in light of the time interval required for the adaptation of a new treatment and the mandatory modifications of clinical workflows. Consequently, we observed an increase of durvalumab application in our clinic from 0% to 85% of eligible patients according to PACIFIC criteria within the first year of drug approval.

The most common reason for ineligibility for durvalumab was disease progression according to RECIST criteria in initial follow-up imaging studies after P-CRT, which affected about one third of patients. Even though the cisplatin-based CRT is currently the best available treatment option for inoperable NSCLC stage III, tumor control rates are only moderate and further research, i.e., in the field of selective radiation dose escalation could possibly contribute to the improvement of this approach (9). In the RTOG 0617 trial, median overall survival for patients who received standard P-CRT was 28.7 months (10).

Nevertheless, the criterion of therapy response to platinum-based CRT for durvalumab eligibility was set arbitrarily in the PACIFIC trial and it is unclear, whether patients with disease progression after P-CRT might likewise benefit from durvalumab therapy.

The second most common reason for durvalumab ineligibility was a PD-L1 TPS of 0% as defined by the EMA approval terms. This affected about 22% of the patients with available PD-L1 expression status in our collective. At first sight it seems to be intuitive that administration of a PD-L1 antibody does not make any sense in a malignancy negative for PD-L1. Nevertheless, it is not finally proven that durvalumab is only effective in PD-L1 positivity of at least 1%. This assumption was based on a retrospective post-hoc analysis of the PACIFIC study group with incomplete data on PD-L1 positivity. Additionally, the tissue samples for PD-L1 testing were taken prior to radiation therapy (11). But, literature demonstrates that PD-L1 is upregulated by RT (12). Hence, effectivity of a PD-L1 antibody can be expected to increase during RT. Furthermore, PD-L1 is tested on a small biopsy in most cases due to inoperability of NSCLC stage III and therefore may not represent the PD-L1 positivity of the entire tumor due to inhomogeneity. Additionally, mechanisms of durvalumab action that are not dependent on initial PD-L1 positivity of tumor cells should be considered. Drug admission boards of other countries like the U.S. Food and Drug Administration (FDA) have approved CIT in NSCLC stage III independent of the degree of PD-L1 expression. Our explorative analysis demonstrates that highly positive PD-L1 TPS (≥50%) was associated to a better chance for CIT eligibility.

The third factor that leads to a relevant number of ineligible patients for CIT is a significant therapy-related toxicity defined as CTCAE ≥ 2. In our study cohort, this affected 15% of patients in first imaging follow-up. All patients who were ineligible for durvalumab due to therapy-related toxicity suffered from pneumonitis. At this point, we also have to discuss that pneumonitis as an exclusion criterion was considered in the PACIFIC trial when it occurred during the first 6 weeks after finishing platinum-based CRT and rates for severe pneumonits of CTCAE ≥ 3 were about 4% (4). A well-researched systematic review demonstrated a rate of severe pneumonitis defined by CTCAE ≥ 3 due to platinum-based CRT of 9%–12% (13). A study that investigated durvalumab eligibility in 81 patients reported a pneumonitis rate CTCAE ≥ 2 of 16% within 6 weeks after finishing platinum-based CRT (5). These data demonstrate that the definition of therapy-related significant pneumonitis differs in severity and timing. Especially the time course of pneumonitis can vary and therefore be challenging to evaluate. It can take several months until first radiologic signs of radiation pneumonitis are detectable. Therefore, the exclusion of CIT on the basis of early radiation pneumonitis remains controversial. On the other hand, late-onset pneumonitis during the course of CIT remains a risk to be considered. The ETOP NICOLAS trial was first to demonstrate that it might be safe to simultaneously combine CRT and anti-PD-L1 antibodies in stage III NSCLC without an increased risk of pneumonitis (14). However, more data from larger-scale ongoing trials is needed in this context.

Other criteria for durvalumab eligibility defined in the PACIFIC trial like autoimmune diseases or uncontrolled comorbidities did contribute to durvalumab withholding in only few patients.

To find influencing factors for durvalumab eligibility, a bootstrap analysis and a logistic regression analysis have been conducted. The analysis emphasizes the time course of therapy as an influencing factor for durvalumab eligibility. Strict adherence to therapy protocol and timelines should be aimed for, as far as medically reasonable and technically possible, so treatment completion can be achieved without major interruptions. This is supported by literature data and proven for several entities including NSCLC (15–19). As descriptive analysis demonstrates, approximately one quarter of patients had a major interruption of radiation therapy. Those were mainly attributed to dose-limiting toxicities or treatment-associated complications in this comorbid study collective. As demonstrated in several phase-III-trials, dose-limiting toxicities can occur frequently during platinum-based CRT. Most frequent among those are pneumonitis, esophagitis, bone marrow suppression, and fatigue (20–23). As the frequency of treatment-associated toxicity has been shown to be associated with the dose distribution of RT, it is especially crucial to focus on adequate dose reduction to sensitive organs at risk during treatment planning. This can be achieved by the use of modern techniques for imaging and RT treatment planning, such as intensity-modulated radiotherapy or PET-based RT planning (24, 25). Some patients withheld compliance for CRT continuation due to therapy-related toxicity. Therapy-related toxicities can severely affect patients’ quality of life and supportive care according to current guidelines is crucial to control these side effects (26). At this point, it should to be mentioned that at first glance the median period of 4 months seems to be quite long from the time of diagnosis to completion of CRT in our study cohort. This comparatively long time period is caused by sequential chemoradiotherapy with induction chemotherapy before radiotherapy start. This is institutional practice to avoid complications while there is no proof for overall superiority of concomitant chemoradiotherapy even if there are data pointing to a better effectivity but also higher toxicity (2, 21, 27). As these patients suffer frequently from comorbidities to our experience in real world the approach of sequential radiochemotherapy is better tolerable.

Another possible influencing factor for durvalumab eligibility was the choice of platinum-containing component of CRT. Patients who received cisplatin seem to have a more than 50% higher probability of being eligible for CIT compared to patients who received carboplatin. In a study cohort of 200 patients suffering from NSCLC stage III, a mild survival benefit of cisplatin and etoposid versus carboplatin and paclitaxel was demonstrated for concomitant CRT (28). Large meta-analyses support this and prove a survival benefit of cisplatin over carboplatin. Nevertheless, the toxicity profile of cisplatin is disadvantageous compared to carboplatin. In cisplatin, nausea and vomiting, neuropathy, nephropathy, alopecia and sensorineural hearing loss are more frequently observed than in carboplatin (29). Therefore, the decision about platinum-containing component of CRT should be made depending on comorbidities, life expectancy and considering patient preferences.

Limitations of this analysis include its retrospective design with its known risk for selection bias, as well as potentially incomplete or contradicting source documentation. PD-L1 expression status was available only for a subgroup of patients, limiting the statistical power of analyses performed only for that respective subgroup.

The study is strengthened by its cohort size and the inclusion of all consecutive stage III patients treated with CRT at a tertiary cancer center of a 10-year period, which allows for an adequate representation of clinical reality. Furthermore, the integrated nature of the dataset allowed for the analysis of detailed information about medical oncologic, as well as radiation oncologic treatment and radiologic follow-up data to asses for initial treatment response. Additionally, our large dataset offered the opportunity to compare eligibility rates and administration rates for durvalumab in our clinic since drug approval in September 2018 in the European Union.

## Conclusion

Our work presents an analysis of a large cohort of consecutive patients from a tertiary cancer center that assesses durvalumab eligibility according to PACIFIC criteria in detail with an in-depth analysis of predictive variables and comparison with real rates of durvalumab administration in clinical practice.

Furthermore, 50.3% of 437 patients who received platinum-based definitive CRT for NSCLC stage III were eligible to receive CIT according to PACIFIC criteria. In addition, 85% of these potentially eligible patients actually received CIT about 1 year after drug approval at a large European tertiary cancer center and the rate of CIT administration showed an increasing trend during that time period. If patients did not meet PACIFIC criteria for durvalumab eligibility, this was mainly caused by disease progression during platinum-based CRT, followed by therapy-related pneumonitis and a PD-L1 expression of 0% as defined by the EMA drug approval terms.

## Data Availability Statement

The raw data supporting the conclusions of this article will be made available by the authors, without undue reservation.

## Ethics Statement

The studies involving human participants were reviewed and approved by Heidelberg University ethics committee on November 20, 2019 (#S-767/2019). Written informed consent for participation was not required for this study in accordance with the national legislation and the institutional requirements.

## Author Contributions

TE, RS, and JD planned and supervised this analysis as part of the pulmonary-radiooncological research group. AK, TM, and NB performed data extraction and review. DW performed all statistical analysis. TE reviewed the data analysis and drafted the manuscript. RS, SR, LD, FB, PC, SK, SA, LK, JH-R, CH, and MT contributed patient data and participated in reviewing and improving the analysis and manuscript. All authors contributed to the article and approved the submitted version.

## Funding

We acknowledge financial support by Deutsche Forschungsgemeinschaft within the funding program for Open Access Publishing, by the Baden-Württemberg Ministry of Science, Research and the Arts, and by Ruprecht-Karls-Universität Heidelberg.

## Conflict of Interest

TE reports grants from Ruprecht-Karls Universität Heidelberg, Herbert Kienzle Foundation, and Else Kröner-Fresenius Foundation and received travel reimbursement from Bristol-Myers Squibb outside the submitted work. JH-R received speaker fees and travel reimbursement from ViewRay Inc, as well as travel reimbursement form IntraOP Medical and Elekta Instrument AB outside the submitted work. SA acknowledges personal fees by Astra Zeneca outside the presented research work. JD reports grants from CRI The Clinical Research Institute, grants from View Ray Inc., grants from Accuray International, grants from Accuray Incorporated, grants from RaySearch Laboratories AB, grants from Vision RT limited, grants from Merck Serono GmbH, grants from Astellas Pharma GmbH, grants from Astra Zeneca GmbH, grants from Siemens Healthcare GmbH, grants from Solution Akademie GmbH, grants from Eromed PLC Surrey Research Park, grants from Quintiles GmbH, grants from Pharmaceutical Research Associates GmbH, grants from Boehringer Ingelheim Pharma GmbH Co, grants from PTW-Freiburg Dr. Pychlau GmbH, and grants from Nanobiotix A.a., outside the submitted work. RS reports grants from Ruprecht-Karls Universität Heidelberg, during the conduct of the study; personal fees from Accuray Inc., personal fees from AstraZeneca GmbH, personal fees from Bristol Myers Squibb GmbH & Co., personal fees from Novocure GmbH, personal fees from Merck KGaA, personal fees from Takeda GmbH, grants from Accuray Inc., outside the submitted work. The other authors declare that the research was conducted in the absence of any commercial or financial relationships that could be construed as a potential conflict of interest.
